# Activation of PPAR*γ* Protects Obese Mice from Acute Lung Injury by Inhibiting Endoplasmic Reticulum Stress and Promoting Mitochondrial Biogenesis

**DOI:** 10.1155/2022/7888937

**Published:** 2022-09-28

**Authors:** Yin Tang, Ke Wei, Ling Liu, Jingyue Ma, Siqi Wu, Wenjing Tang

**Affiliations:** Department of Anesthesiology, The First Affiliated Hospital of Chongqing Medical University, Chongqing 400016, China

## Abstract

**Objective:**

Obesity-induced endoplasmic reticulum (ER) stress plays a role in increased susceptibility to acute lung injury (ALI)/acute respiratory distress syndrome (ARDS). The activation of peroxisome proliferator-activated receptor-*γ* (PPAR*γ*) is associated with lung protection and is effective in ameliorating ER stress and mitochondrial dysfunction. The aim of this study was to investigate the expression of PPAR*γ* in the lung tissues of obese mice and explore whether the PPAR*γ*-dependent pathway could mediate decreased ALI/ARDS by regulating ER stress and mitochondrial biogenesis.

**Methods:**

We determined PPAR*γ* expression in the lung tissues of normal and obese mice. ALI models of alveolar epithelial cells and of obese mice were used and treated with either PPAR*γ* activator rosiglitazone (RSG) or PPAR*γ* inhibitor GW9662. Lung tissue and cell samples were collected to assess lung inflammation and apoptosis, and ER stress and mitochondrial biogenesis were detected.

**Results:**

PPAR*γ* expression was significantly decreased in the lung tissue of obese mice compared with that in normal mice. Both in vivo and in vitro studies have shown that activation of PPAR*γ* leads to reduced expression of the ER stress marker proteins 78-kDa glucose-regulated protein (GRP78), C/EBP homologous protein (CHOP), and Caspase12. Conversely, expression of the mitochondrial biogenesis-related proteins peroxisome proliferator-activated receptor *γ* coactivator 1 (PGC-1*α*), nuclear respiratory factor-1 (NRF-1), and mitochondrial transcription factor A (TFAM) increased. Furthermore, activation of PPAR*γ* is associated with decreased levels of lung inflammation and epithelial apoptosis and increased expression of adiponectin (APN) and mitofusin2 (MFN2). GW9662 bound to PPAR*γ* and blocked its transcriptional activity and then diminished the protective effect of PPAR*γ* on lung tissues.

**Conclusions:**

PPAR*γ* activation exerts anti-inflammation effects in alveolar epithelia by alleviating ER stress and promoting mitochondrial biogenesis. Therefore, lower levels of PPAR*γ* in the lung tissues of obese mice may lead to an increased susceptibility to ALI.

## 1. Introduction

The dramatic rise in the global prevalence of obesity is a growing health concern that seriously affects the quality of life and reduces life expectancy [1]. A series of characteristic comorbidities have been linked with obesity, including sleep apnea, diabetes, hypertension, hyperlipidemia, and heart disease [2]. It was also recognized that obesity is a chronic inflammatory state, which is associated with increased risk of acute lung injury (ALI) or even acute respiratory distress syndrome (ARDS) [3, 4]. The mechanism underlying the aggravation of ALI in obese individuals has not yet been clarified. Current evidence has attributed this susceptibility to ALI due to disturbances in adipocytokine secretion or aberrant endoplasmic reticulum (ER) stress [5, 6].

>Peroxisome proliferator-activated receptors (PPARs) are ligand-activated transcription factors that are members of the nuclear receptor superfamily. The PPAR superfamily comprises three subtypes, PPAR*α*, PPAR*γ*, and PPAR*β*/*δ*, with differential tissue distribution [7]. As one of the three PPAR subtypes, PPAR*γ* can be expressed in alveolar epithelial cells, vascular endothelial cells, and macrophages [8–11]. PPAR*γ* is involved in processes such as adipogenesis, insulin sensitivity, mitochondrial biogenesis, anti-inflammation, and neuroprotection [12–15]. In addition, PPAR*γ* plays a protective role in ALI [16], lung cancer [17], chronic obstructive pulmonary disease, and other respiratory diseases [18]. PPAR*γ* ligands include synthetic and natural ligands. Synthetic ligands include thiazolidinedione antidiabetic drugs such as RSG, pioglitazone, and troglitazone. Among them, RSG is a representative PPAR*γ* agonist [19]. PPAR*γ* is present in both the cytoplasm and the nucleus. Ligand-activated PPAR*γ* regulates target genes through heterodimerization with retinoid X receptor (RXR) [20]. GW9662 is a specific antagonist of PPAR*γ* that covalently binds to the PPAR*γ* ligand-binding pocket, preventing the activation of ligand binding and disrupting PPAR*γ* signaling [21].

Activation of PPAR*γ* can reduce the release of inflammatory factors, but its expression is always inhibited during ALI [22]. To date, the level of PPAR*γ* in the lung tissues of obese mice is still unknown, nor is its role in the progression of ALI. PPAR*γ* is beneficial for promoting mitochondrial biogenesis and inhibiting ER stress, a prominent feature associated with diabetes, obesity, and chronic inflammation [23–25]. Previous studies have suggested that ER stress and mitochondrial dysfunction play key roles in mediating lung injury in obese mice [6, 26]. In this study, we hypothesized that PPAR*γ* activation can protect obese mice against ALI by regulating ER stress and mitochondrial biogenesis. Accordingly, we investigated the expression of PPAR*γ* in the lung tissues of obese mice and explored the role of PPAR*γ* in LPS-induced injury in lung tissues and pulmonary epithelial cells.

## 2. Methods

### 2.1. Experimental Animals

The animal experimental protocol was approved by the Animal Ethics Committee of the First Affiliated Hospital of Chongqing Medical University (2021-527). This experiment was performed in accordance with the Guidelines for the Care and Use of Experimental Animals. Six-week-old male C57BL/6 J mice, weighing 15-18 g, were purchased from the Experimental Animal Center of Chongqing Medical University. During the study, all mice were kept on a 12/12 h light/dark cycle. The animals were fed either a normal chow diet or a high-fat diet with 60% calories from fat (TP2330055A, Trophic Animal Feed High-tech Co., Ltd., China) for 12 weeks. In this study, every effort was made to minimize distress in mice.

The mice were anesthetized by sodium pentobarbital (50 mg/kg) (Sigma-Aldrich), injected intraperitoneally [16]. ALI was induced by administering 100 *μ*g (1 mg/mL) of lipopolysaccharide (LPS, L8880, Solarbio, Beijing, China) into the trachea of anesthetized mice and gently pulling out the tongue with forceps to facilitate fluid entry into the lungs [6]. Both PPAR*γ* activator RSG (HY-17386, MedChemExpress, Shanghai, China) and PPAR*γ* inhibitor GW9662 (HY-116578, MedChemExpress) were dissolved in 10% dimethyl sulfoxide (DMSO; D8371, Solarbio, Beijing, China). RSG and GW9662 were injected intraperitoneally at respective doses of 10 mg/kg and 1 mg/kg. The RSG and GW9662 doses were determined based on previous studies [16, 27, 28]. An equal volume of DMSO was injected intraperitoneally as a vehicle control for all mouse experiments.

### 2.2. Animal Experimental Design

**Figure 1:**
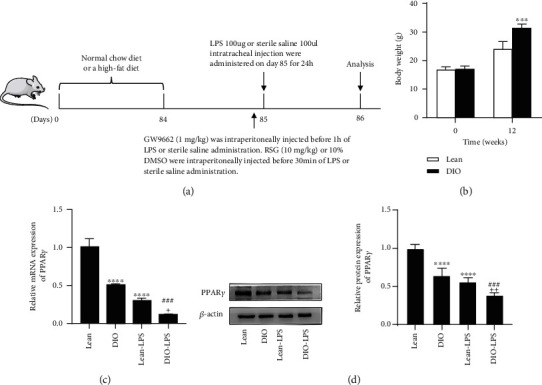
PPAR*γ* expression was decreased in the lung tissue of obese mice. (a) Schematic diagram of experimental protocol, (b) Body weight of C57BL/6 J mice fed with either high fat diet or normal diet for 12 weeks, (c) Relative mRNA expression of PPAR*γ*. (d) Western blotting and relative protein expression of PPAR*γ*. Data are presented as mean ± SD. ^∗∗∗^*p* < 0.001, ^∗∗∗∗^*p* < 0.0001*vs.* Lean, ^###^*p* < 0.001*vs.* DIO, ^+^*p* < 0.05, ^++^*p* < 0.01*vs.* Lean-LPS.

According to our experimental plan, the mice were divided into the following 8 groups (*n* = 5): (1) lean group (normal diet mice): 100 *μ*L sterile saline administered intratracheally; (2) DIO group (diet-induced obese mice): 100 *μ*L of sterile saline administered intratracheally; (3) lean-ALI group: LPS (100 *μ*g) administered intratracheally; (4) in the DIO-ALI group, mice were administered 100 *μ*g LPS intratracheally; (5) DIO-DMSO group: mice were injected intraperitoneally with 10% DMSO, and 30 min later, 100 *μ*L sterile saline was introduced in the mouse trachea; (6) in the DIO-ALI-DMSO group, mice were intraperitoneally injected with 10% DMSO, and 30 min later, 100 *μ*g of LPS was introduced in the mouse trachea; (7) in the DIO-ALI-RSG group, mice were intraperitoneally injected with RSG (10 mg/kg), and 30 min later, 100 *μ*g of LPS was introduced in the mouse trachea; and(8) DIO-ALI-RSG-GW9662 group: mice were treated the same as in the DIO-ALI-RSG group, but were intraperitoneally injected with GW9662 (1 mg/kg) 30 min before administration of RSG, while the other groups received the same amount of normal saline. A schematic of the experimental protocol is shown in Figure 1(a).

### 2.3. Collection of Bronchoalveolar Lavage Fluid (BALF)

The mice were euthanized at 24 h after LPS treatment, and the right main bronchus was ligated. The left lung was douched by administering sterile saline (3 × 0.5 ml) into the trachea. The collected BALF was centrifuged at 200 × *g* for 10 min to separate the pellet and supernatant, and the supernatant so obtained was stored at − 80 °C. The levels of tumor necrosis factor-alpha (TNF-*α*) and Interleukin-1*β* (IL-1*β*) in BALF were determined using an enzyme-linked immunosorbent assay (ELISA) kit (Neobioscience Technology Company, Shenzhen, China) according to the manufacturer's instructions.

### 2.4. Lung Wet/Dry (W/D) Weight Ratio

The right parietal lobe lung tissue was weighed to determine the wet weight. The sample was dried in an oven at 80 °C for 48 h, and was weighed again to obtain the dry weight. Finally, the W/D of the lung was obtained.

### 2.5. Histopathology

The lower lobe of the right lung was fixed in 4% paraformaldehyde and embedded in paraffin. Paraffin-embedded tissue sections were stained with hematoxylin and eosin (H&E). Alveolar edema, hemorrhage, alveolar septal thickening, and infiltrating leukocyte count were recorded to assess the extent of lung injury. Each of these histopathological elements was allotted four grades from 0 to 3 (0 = normal, 1 = mild, 2 = moderate, and 3 = severe) [29].

### 2.6. Immunohistochemical Staining

Immunohistochemical staining was performed as follows. Paraffin-embedded specimens were sectioned at 5 *μ*m, deparaffinized, and hydrated. The sections were then incubated with 3% H_2_O_2_ for 10 min and rinsed with phosphate-buffered saline (PBS). Primary antibodies against GRP78 (ab21685, Abcam) and PGC-1*α* (ab191838, Abcam) were used. The slides were then washed and incubated with secondary antibodies at room temperature for 30 min. The sections were observed under an optical microscope.

### 2.7. TUNEL Staining

To evaluate the LPS-induced apoptosis of alveolar epithelial cells in obese mice, terminal deoxynucleotidyl transferase-mediated dUTP nick end labeling (TUNEL) staining was performed according to the manufacturer's instructions (G1507, Servicebio, Wuhan, China).

### 2.8. Cell Culture and Treatment

A549 human lung epithelial cells were used to study the effects of PPAR*γ* on alveolar epithelial cells. The A549 cell line is commonly used to study inflammation and apoptosis induced by LPS [30, 31], and PPAR*γ* has been shown to be expressed in this cell line [32, 33]. A549 cells were seeded into plastic petri dishes with RPMI 1640 medium (C11875500BT, Gibco, USA) containing 10% fetal bovine serum (10099-141C, Gibco, USA), 100 U/ml penicillin, and 0.1 mg/ml streptomycin (SV30010, HyClone, USA) and placed at 37 °C with 5% CO_2_ in the incubator. The cells were divided into four groups: control, LPS, LPS-RSG, and LPS-RSG-GW9662. The LPS group was treated with 10 *μ*g/ml LPS. In the LPS-RSG group, 10 *μ*M RSG was added 30 min before the addition of LPS. In the LPS-RSG-GW9662 group, 20 *μ*M GW9662 was added 30 min before adding RSG. An equal volume of PBS was added to the control group. Doses of these drugs were determined based on previous studies [12, 23, 34, 35]. RSG and GW9662 were incubated in vitro in a final concentration of 0.1% DMSO. At 24 h after LPS treatment, the cells were collected for further experiments.

### 2.9. Cytokines Assays

Cell culture supernatants were collected, added to sterile tubes, and centrifuged at 560 × *g* for 10 min. The supernatant so collected was stored at − 80 °C. The levels of TNF-*α* and IL-1*β* in cell culture supernatants were determined using an ELISA kit.

### 2.10. Immunofluorescence

The expression of PGC-1*α* and GRP78 in A549 cells was detected using immunofluorescence. Coverslips bearing A549 cells were collected from each group. The cells were fixed and permeabilized with 4% paraformaldehyde and 0.5% Triton X-100. Cells were blocked with 1% normal goat serum (Beyotime Biotechnology, Shanghai, China) for 30 min at room temperature and then incubated with primary antibodies PGC-1*α* (1 : 400) and GRP78 (1 : 200) overnight at 4 °C. Cy3-conjugated Affinipure Goat anti-rabbit IgG (H + L) (SA00009-2, Proteintech, Wuhan, China) was used as a secondary antibody and incubated for 1 h at room temperature in the dark. The cells were then fixed with an antifade mounting medium containing DAPI (P0131, Beyotime Biotechnology, Shanghai, China). Photomicrographs were obtained using a confocal microscope (ZEISS, Oberkochen, Germany).

### 2.11. Flow Cytometric Analysis

The cells were harvested by trypsinization and washed twice with cold PBS. Apoptotic cells were quantified using an annexin V-FITC detection kit (Elabscience, Wuhan, China). We performed flow cytometry (Cytoflex, USA) and analyzed the results using the Cytoflex software. The total proportion of apoptotic cells was calculated by adding the numbers of late and early apoptotic cells.

### 2.12. Quantitative Real-Time PCR

Total RNA was isolated from lung tissue and A549 cells using TRIzol reagent (Takara Biotechnology). Total RNA was reverse-transcribed into cDNA using a reverse transcription kit (HY-K0511A; MedChemExpress, Shanghai, China). PCR amplification was quantified using SYBR Green qPCR Master Mix (HY-K0523; MedChemExpress, Shanghai, China). *β*-Actin was used as an internal control. Primers used in this study are listed in Table 1.

### 2.13. Western Blotting

A549 cells and lung tissue samples were collected, homogenized, and lysed using RIPA lysis buffer (P0013B, Beyotime Biotechnology, Shanghai, China) containing protease and phosphatase inhibitors (P1045, Beyotime Biotechnology, Shanghai, China) for 30 min on ice. After centrifugation at 12000 × *g* for 15 min at 4 °C, the supernatant was collected, and the protein concentrations were detected using the BCA method (P0010, Beyotime Biotechnology, Shanghai, China). Proteins were separated on SDS-PAGE gels and transferred to polyvinylidene fluoride (PVDF) membranes (Millipore, Billerica, MA, USA), which were blocked with 5% non-fat milk for 1 h at room temperature and then incubated overnight at 4 °C with primary antibodies against PPAR*γ* (2435, Cell Signaling Technology), PGC-1*α* (ab191838, Abcam), NRF-1 (ab175932, Abcam), TFAM (ab47517, Abcam), MFN2 (ab124773, Abcam), GRP78 (ab21685, Abcam), CHOP (ab11419, Abcam), Caspase12 (ab8118, Abcam), Caspase3 (9662, Cell Signaling Technology), APN (21613-1-AP, Proteintech), and *β*-actin (20536-1-AP, Proteintech). After washing thrice, the membranes were incubated with secondary antibodies (SA00001-2, Proteintech) for 1 h at room temperature. The relative intensities of the protein bands were analyzed using the Bio-Rad Quantity One software. The results were normalized to *β*-actin levels.

### 2.14. Statistics Analysis

All data are expressed as the mean ± SD. Statistical analyses were performed using the GraphPad Prism 8.0 software (GraphPad Software, USA). The Student's *t*-test was used for two-group comparisons, and one-way ANOVA was used for multiple-group comparisons. In all analyses, statistical significance was set at *p* < 0.05.

## 3. Results

### 3.1. Activation of PPAR*γ* Attenuated LPS-Induced Apoptosis and Inflammation in Alveolar Epithelial Cells

**Figure 2:**
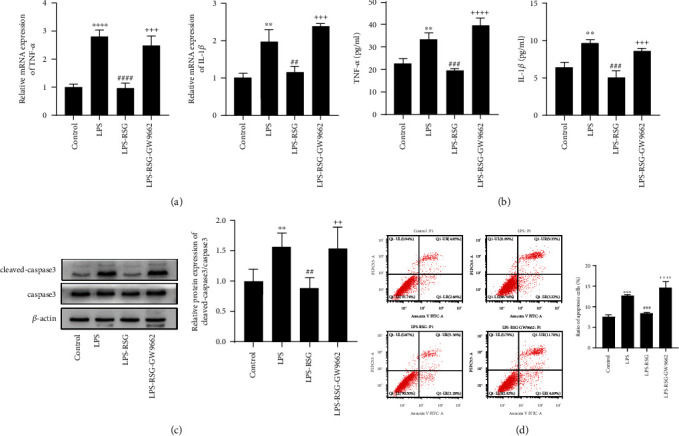
Activation of PPAR*γ* attenuated LPS-induced apoptosis and inflammation in alveolar epithelial cells. (a) Relative mRNA expressions of TNF-*α* and IL-1*β* in A549 epithelial cells; (b) Levels of TNF-*α* and IL-1*β* in cell culture supernatants; (c) Western blotting and relative protein expression of cleaved-caspase3/caspase3 in A549 alveolar epithelial cell (cleaved-caspase3 was an activated form of caspase3); and (d) flow cytometry was used to detect the apoptosis of A549 alveolar epithelial cells and the percentage of apoptosis of A549 alveolar epithelial cells. Data are presented as mean ± SD.^∗∗^*p* < 0.01, ^∗∗∗^*p* < 0.001, ^∗∗∗∗^*p* < 0.0001*vs.* Control, ^##^*p* < 0.01, ^###^*p* < 0.001, ^####^*p* < 0.0001*vs.* LPS, ^+++^*p* < 0.001, ^++++^*p* < 0.0001*vs.* LPS-RSG.

To confirm the effect of PPAR*γ* on alveolar epithelial cells, we selected human type II alveolar epithelial cells A549 for the in vitro experiments. Our results showed that LPS significantly increased inflammation and apoptosis in alveolar epithelial cells (Figure 2). Treatment with RSG significantly decreased the LPS-induced expression of TNF-*α* and IL-1*β* in alveolar epithelial cells (Figures 2(a) and 2(b)). Western blotting showed that RSG treatment reduced the cleavage of caspase 3 (Figure 2(c)), and flow cytometry results also showed that RSG administration significantly reduced the rate of apoptosis and necrosis in A549 cells (Figure 2(d)). However, we found that RSG-stimulated changes were prevented by GW9662 (Figure 2), indicating that these effects were mediated by the PPAR*γ* signaling pathway. Our results demonstrate that PPAR*γ* activation attenuates LPS-induced apoptosis and inflammation in alveolar epithelial cells.

### 3.2. Activation of PPAR*γ* Inhibited LPS-Induced ER Stress in Alveolar Epithelial Cells

**Figure 3:**
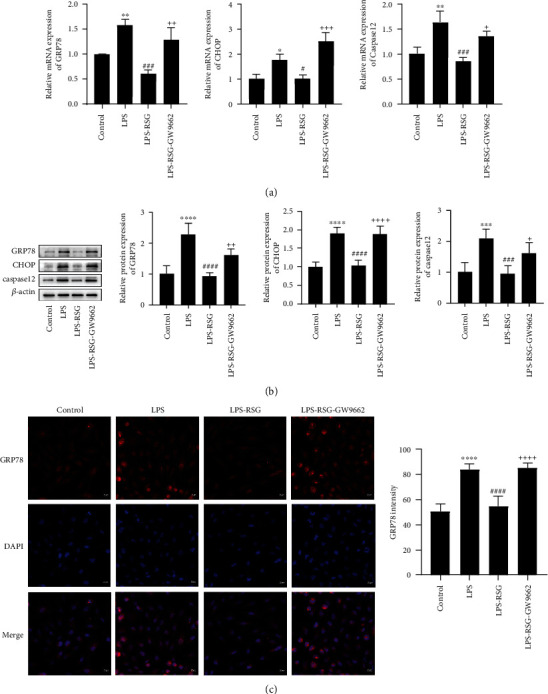
Activation of PPAR*γ* inhibited LPS-induced ER stress in alveolar epithelial cells. (a) Relative mRNA expressions of GRP78, CHOP, and Caspase12 in A549 epithelial cells. (b) Western blotting and relative protein expressions of GRP78, CHOP, and Caspase12 in A549 epithelial cells. (c) Immunofluorescence staining and mean fluorescence intensity of GRP78 in A549 epithelial cells (original magnification, ×200). Data are presented as mean ± SD. ^∗^*p* < 0.05, ^∗∗^*p* < 0.01, ^∗∗∗^*p* < 0.001, ^∗∗∗∗^*p* < 0.0001*vs.* Control, ^#^*p* < 0.05, ^###^*p* < 0.001, ^####^*p* < 0.0001*vs.* LPS, ^+^*p* < 0.05, ^++^*p* < 0.01, ^+++^*p* < 0.001, ^++++^*p* < 0.0001*vs.* LPS-RSG.

To investigate whether PPAR*γ* directly reduced ER stress in alveolar epithelial cells, we measured the expression of ER stress-related indicators in alveolar epithelial cells. The results showed that LPS significantly upregulated the expression of ER stress-related proteins (Figure 3). Administration of RSG suppressed the mRNA and protein expressions of GRP78, CHOP, and Caspase12 in alveolar epithelial cells (Figures 3(a) and 3(b)). Immunofluorescence analysis showed that RSG decreased the expression of the ER stress marker protein GRP78 in alveolar epithelial cells (Figure 3(c)). The PPAR*γ*-specific blocker, GW9662, prevented the effects of RSG (Figure 3). These results indicate that PPAR*γ* activation could inhibit LPS-induced ER stress in alveolar epithelial cells.

### 3.3. Activation of PPAR*γ* Promoted Mitochondrial Biogenesis in Alveolar Epithelial Cells

**Figure 4:**
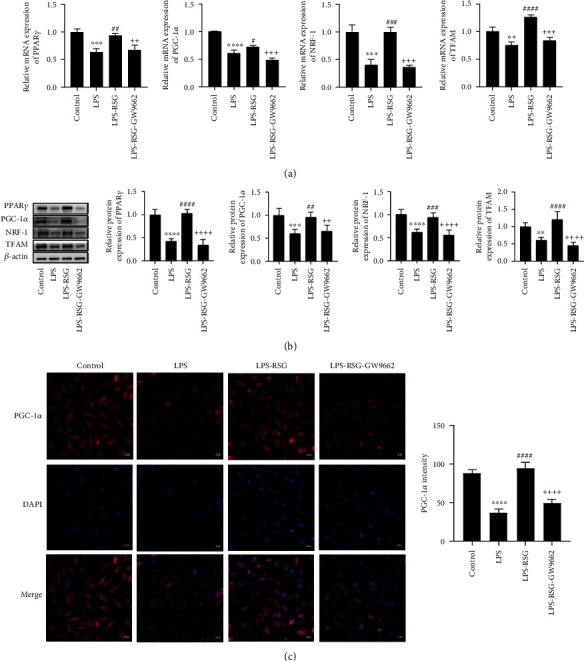
Activation of PPAR*γ* promoted mitochondrial biogenesis in alveolar epithelial cells. (a) Relative mRNA expressions of PPAR*γ*, PGC-1*α*, NRF-1, and TFAM in A549 epithelial cells. (b) Western blotting and relative protein expressions of PPAR*γ*, PGC-1*α*, NRF-1, and TFAM in A549 epithelial cells. (c) Immunofluorescence staining and mean fluorescence intensity of PGC-1*α* in A549 epithelial cells (original magnification, ×200). Data are presented as mean ± SD. ^∗∗^*p* < 0.01, ^∗∗∗^*p* < 0.001, ^∗∗∗∗^*p* < 0.0001*vs.* Control, ^#^*p* < 0.05, ^##^*p* < 0.01, ^###^*p* < 0.001, ^####^*p* < 0.0001*vs.* LPS, ^++^*p* < 0.01, ^+++^*p* < 0.001, ^++++^*p* < 0.0001*vs.* LPS-RSG.

To investigate the effect of PPAR*γ* on mitochondrial biogenesis in alveolar epithelial cells, we examined the expression of mitochondrial biogenesis-related indicators in alveolar epithelial cells. The results showed that LPS significantly decreased the expression of mitochondrial biogenesis-related proteins (Figure 4). However, the administration of RSG suppressed the damaging effects of LPS on mitochondrial biogenesis. RSG upregulated the mRNA and protein expressions of PPAR*γ*, PGC-1*α*, NRF-1, and TFAM (Figures 4(a) and 4(b)). Immunofluorescence also showed that RSG increased the expression of the mitochondrial biogenesis marker protein PGC-1*α* in alveolar epithelial cells (Figure 4(c)). However, RSG-stimulated changes were prevented by GW9662 treatment (Figure 4). These results suggest that PPAR*γ* activation can promote mitochondrial biogenesis in alveolar epithelial cells.

### 3.4. Activation of PPAR*γ* Promoted MFN2 Expression in Alveolar Epithelial Cells

**Figure 5:**
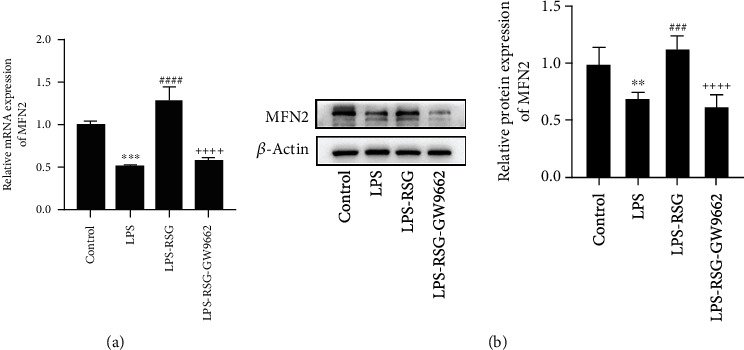
Activation of PPAR*γ* promoted MFN2 expression in alveolar epithelial cells. (a) Relative mRNA expression of MFN2, (b) Western blotting and relative protein expression of MFN2. Data are presented as mean ± SD. ^∗∗^*p* < 0.01, ^∗∗∗^*p* < 0.001*vs.* Control; ^###^*p* < 0.001, ^####^*p* < 0.0001*vs.* LPS; ^++++^*p* < 0.0001*vs.* LPS-RSG.

Mfn2 is embedded in the mitochondrial outer membrane. It mediates mitochondrial fusion by the tethering of two adjacent mitochondria and is involved in the connection between mitochondria and the ER. Our study showed that LPS inhibited the expression of MFN2 in alveolar epithelial cells. Administration of RSG can promote the expression of MFN2, but the effect of RSG was prevented by GW9662 (Figure 5). Our results suggested that MFN2 expression is regulated by a PPAR*γ*-dependent pathway.

### 3.5. PPAR*γ* Expression Was Decreased in the Lung Tissue of Obese Mice

To determine the expression of PPAR*γ* in the lung tissue of obese mice, we constructed an obese mouse model and detected the expression of PPAR*γ* in the lung tissue. After 12 weeks, both the groups gained weight. Mice fed the high-fat diet had a significantly higher body weight than that of mice fed the normal diet (Figure 1(b)). Our results showed that the mRNA and protein expression levels of PPAR*γ* in the DIO and DIO-LPS groups were lower than those in the lean and lean-LPS groups. Compared to those in the lean and DIO groups, there was a significant decrease in lung PPAR*γ* mRNA and protein expression in the lean-LPS and DIO-LPS groups (Figures 1(c) and 1(d)). Our study showed that obesity can lead to decreased PPAR*γ* expression in lung tissue and LPS intervention can aggravate this decrease in PPAR*γ* expression.

### 3.6. Activation of PPAR*γ* Attenuated LPS-Induced Lung Injury in Obese Mice

**Figure 6:**
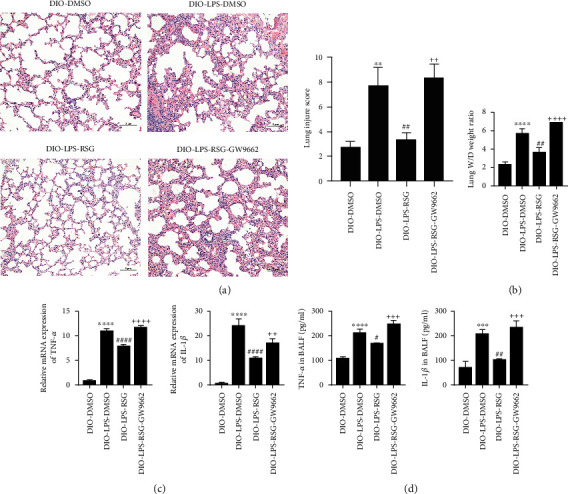
Activation of PPAR*γ* attenuated LPS-induced lung injury in obese mice. (a) HE-stained sections of lung tissue and lung injure score (original magnification, ×200). (b) Wet/dry weight ratio of lungs. (c) Relative mRNA expressions of TNF-*α* and IL-1*β* in lung tissue. (d) Determination of TNF-*α* and IL-1*β* levels in BALF by ELISA. Data are presented as mean ± SD. ^∗∗^*p* < 0.01, ^∗∗∗^*p* < 0.001, ^∗∗∗∗^*p* < 0.0001*vs.* DIO-DMSO, ^#^*p* < 0.05,^##^*p* < 0.01, ^####^*p* < 0.0001*vs.* DIO-LPS-DMSO, ^++^*p* < 0.01, ^+++^*p* < 0.001, ^++++^*p* < 0.0001*vs.* DIO-LPS-RSG.

To evaluate the protective effect of PPAR*γ* on ALI in obese mice, we performed HE staining of lung tissue, lung W/D weight ratio measurements, and detection of inflammatory factors in lung tissue and BALF. Our results showed that the administration of RSG attenuated LPS-induced lung tissue damage. HE staining results showed that RSG decreased LPS-induced inflammatory cell infiltration, hemorrhage, interstitial edema, and alveolar septal thickening in the lung tissue of obese mice (Figure 6(a)). At the same time, RSG diminished the lung W/D weight ratio of obese mice (Figure 6(b)), and the mRNA expression and levels of TNF-*α* and IL-1*β* in lung tissue and BALF decreased (Figures 6(c) and 6(d)). However, the effects of RSG were inhibited by GW9662 treatment (Figure 6). These observations suggested that PPAR*γ* activation protects obese mice from ALI.

### 3.7. Activation of PPAR*γ* Inhibited LPS-Induced ER Stress in the Lung Tissue of Obese Mice

**Figure 7:**
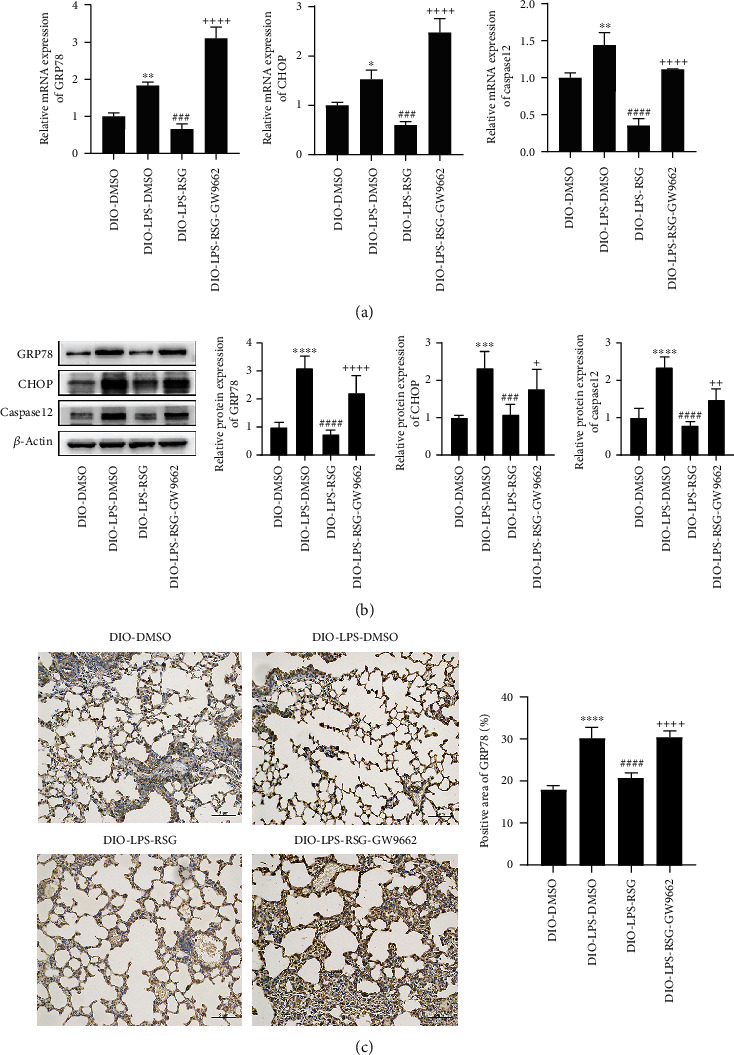
Activation of PPAR*γ* inhibited LPS-induced ER stress in lung tissue of obese mice. (a) Relative mRNA expressions of GRP78, CHOP, and Caspase12 in lung tissue. (b) Western blotting and relative protein expressions of GRP78, CHOP, and Caspase12 in lung tissue. (c) Immunohistochemical staining and positive area ratio of GRP78 in lung tissue (original magnification, ×200). Data are presented as mean ± SD. ^∗^*p* < 0.05, ^∗∗^*p* < 0.01, ^∗∗∗^*p* < 0.001, ^∗∗∗∗^*p* < 0.0001*vs.* DIO-DMSO, ^###^*p* < 0.001, ^####^*p* < 0.0001*vs.* DIO-LPS-DMSO, ^+^*p* < 0.05, ^++^*p* < 0.01, ^++++^*p* < 0.0001*vs.* DIO-LPS-RSG.

To investigate the effects of PPAR*γ* on ER stress in ALI obese mice, we detected the expression of ER stress-related proteins in lung tissue. The results showed that LPS significantly upregulated the expression of ER stress-related proteins (Figure 7). Administration of RSG decreased the mRNA and protein expression of GRP78, CHOP, and Caspase12 (Figures 7(a) and 7(b)). At the same time, immunohistochemistry showed that RSG reduced the expression of GRP78 in lung tissue (Figures 7(c)). However, GW9662 prevented these effects of RSG (Figure 7). These results are consistent with those of the cell studies. Therefore, our results confirmed that PPAR*γ* activation could inhibit LPS-induced ER stress in the lung tissue of obese mice.

### 3.8. Activation of PPAR*γ* Promoted Mitochondrial Biogenesis in the Lung Tissue of ALI Obese Mice

**Figure 8:**
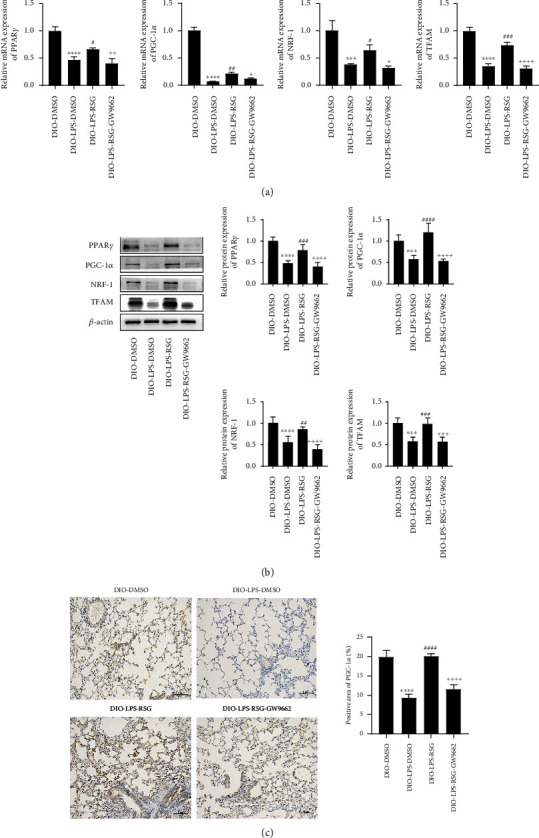
Activation of PPAR*γ* promoted mitochondrial biogenesis in lung tissue of ALI obese mice. (a) Relative mRNA expressions of PPAR*γ*, PGC-1*α*, NRF-1, and TFAM in lung tissue. (b) Western blotting and relative protein expressions of PPAR*γ*, PGC-1*α*, NRF-1, and TFAM in lung tissue. (c) Immunohistochemical staining and positive area ratio of PGC-1*α* in lung tissue (original magnification, ×200). Data are presented as mean ± SD. ^∗∗∗^*p* < 0.001, ^∗∗∗∗^*p* < 0.0001*vs.* DIO-DMSO, ^#^*p* < 0.05, ^##^*p* < 0.01, ^###^*p* < 0.001, ^####^*p* < 0.0001*vs.* DIO-LPS-DMSO, ^+^*p* < 0.05, ^++^*p* < 0.01, ^+++^*p* < 0.001, ^++++^*p* < 0.0001*vs.* DIO-LPS-RSG.

Since the activation of PPAR*γ* promotes mitochondrial biogenesis in alveolar epithelial cells, we speculated that PPAR*γ* also has a positive effect in ALI obese mice. We examined the expression of mitochondrial biogenesis-related markers in the lung tissue. The results showed that, compared with the DIO-LPS-DMSO group, the mRNA and protein expression levels of PPAR*γ*, PGC-1*α*, NRF-1, and TFAM were significantly increased in the DIO-LPS-RSG group (Figures 8(a) and 8(b)). Immunohistochemistry also showed that RSG upregulated the expression of the mitochondrial biogenesis marker protein PGC-1*α* in lung tissue (Figure 8(c)). The effects of RSG were prevented by GW9662 treatment (Figure 8). These results were also consistent with those of the cell studies, and they suggest that PPAR*γ* activation can promote mitochondrial biogenesis in the lung tissue of mice with ALI.

### 3.9. Activation of PPAR*γ* Inhibited LPS-Induced Apoptosis in Lung Tissue of Obese Mice

**Figure 9:**
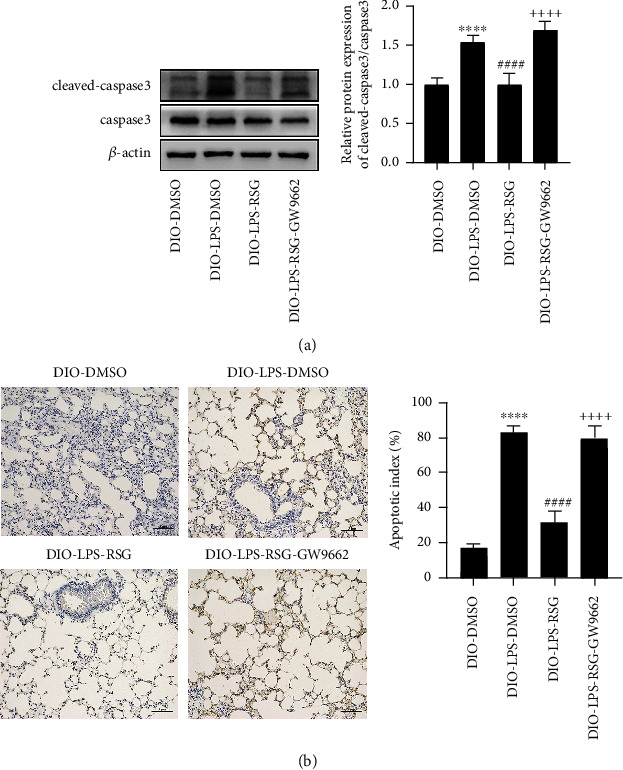
Activation of PPAR*γ* inhibited LPS-induced apoptosis in lung tissue of obese mice. (a) Western blotting and relative protein expression of cleaved-caspase3/caspase3 in lung tissue (cleaved-caspase3 was an activated form of caspase3). (b) TUNEL staining was used to detect apoptosis and apoptotic index of alveolar epithelial cells (original magnification, ×200). Data are presented as mean ± SD. ^∗∗∗∗^*p* < 0.0001*vs.* DIO-DMSO; ^####^*p* < 0.0001*vs.* DIO-LPS-DMSO; ^++++^*p* < 0.0001*vs.* DIO-LPS-RSG.

We explored the protective effects of PPAR*γ* activation on lung tissue cells in obese mice. LPS significantly upregulated the cleavage of caspase 3 in lung tissue, and RSG administration reduced the cleavage of caspase 3; however, GW9662 prevented this effect (Figures 9(a)). TUNEL staining showed that LPS-induced apoptosis of alveolar epithelial cells was significantly increased in obese mice. Administration of RSG reduced alveolar epithelial cell apoptosis in TUNEL-stained lung sections, but this effect was prevented by GW9662 (Figure 9(b)). This is consistent with our in vitro study on the protective effect of PPAR*γ* on alveolar epithelial cells. Our results demonstrated that PPAR*γ* activation inhibited LPS-induced apoptosis of alveolar epithelial cells and protected against ALI in obese mice.

### 3.10. Activation of PPAR*γ* Promoted APN and MFN2 Expression in Lung Tissue of Obese Mice

**Figure 10:**
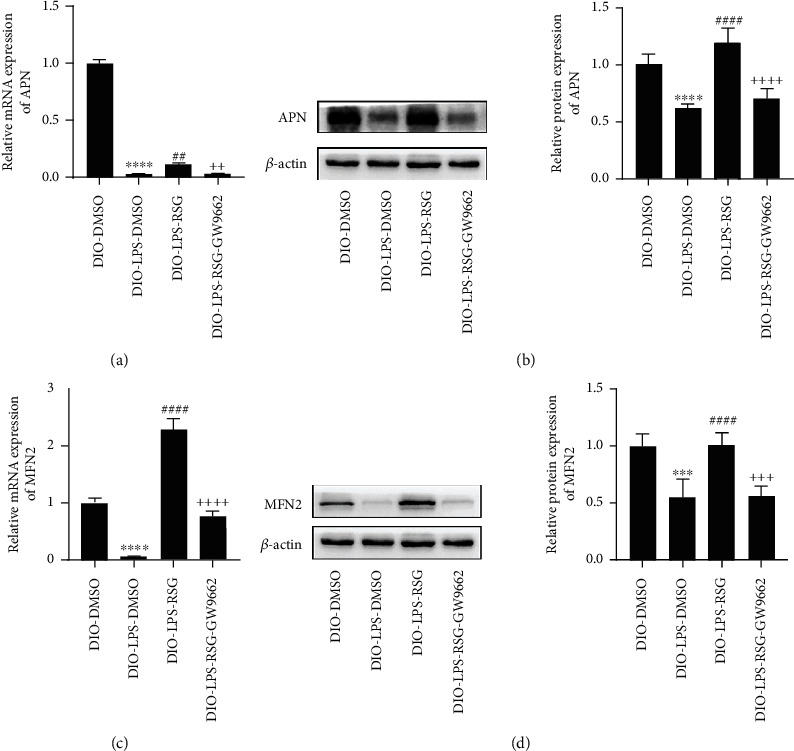
Activation of PPAR*γ* promoted APN and MFN2 expression in lung tissue of obese mice. (a) Relative mRNA expression of APN in lung tissue. (b) Western blotting and relative protein expression of APN in lung tissue. (c) Relative mRNA expression of MFN2 in lung tissue. (d) Western blotting and relative protein expression of MFN2 in lung tissue. Data are presented as mean ± SD. ^∗∗∗^*p* < 0.001, ^∗∗∗∗^*p* < 0.0001*vs.* DIO-DMSO; ^##^*p* < 0.01, ^####^*p* < 0.0001*vs.* DIO-LPS-DMSO; ^++^*p* < 0.01, ^+++^*p* < 0.001, ^++++^*p* < 0.0001*vs.* DIO-LPS-RSG.

To investigate the effect of PPAR*γ* on APN and MFN2 expression, we examined APN and MFN2 expression in the lung tissues of obese mice. Our study showed that LPS reduced the mRNA and protein expression of APN and MFN2 in the lung tissue of obese mice. Administration of RSG promoted APN and MFN2 expression; however, the effect of RSG was prevented by GW9662 (Figure 10). Our results suggest that APN and MFN2 expression are regulated by a PPAR*γ*-dependent pathway.

## 4. Discussion

In this study, several observations were made to improve our current understanding of the role of PPAR*γ*-dependent pathway in ALI. We found that obesity was associated with lower PPAR*γ* expression in lung tissues and its level further decreased during ALI. Our study in alveolar epithelial cells suggests that activation of PPAR*γ*/PGC-1*α* is beneficial for relieving inflammation and apoptosis, accompanied by improved mitochondrial biogenesis and reduced ER stress. In vivo studies in obese mice have also confirmed the protective effect of PPAR*γ* against ALI. Moreover, PPAR*γ* can induce the elevated production of APN and MFN2 in lung tissues, which may facilitate its anti-inflammatory efficacy.

PGC-1*α* is a master regulator of mitochondrial biogenesis and was originally discovered in brown adipose tissue as a co-activator of PPAR*γ* [12]. It can activate PPAR*γ* target genes by inducing the binding of PPAR*γ* ligands to PPAR*γ* [36]. In addition, PGC-1*α* gene expression is a direct target of PPAR*γ* activation. The presence of a PPAR*γ*-responsive element in the distal region of the PGC-1*α* gene promoter binds PPAR*γ*/RXR heterodimers [37]. Previous studies have demonstrated the role of the PPAR*γ*/PGC-1*α* pathway in inhibiting obesity, delaying the progression of chronic obstructive pulmonary disease, and attenuating pulmonary edema [18, 38, 39]. The present study confirmed the role of PGC-1*α* in mediating the protective effects of PPAR*γ* on alveolar epithelial cells. It is reasonable to assume that the decreased activity of PPAR*γ*/PGC-1*α* in obese individuals may prompt the lung to exacerbate injuries.

Impairment of mitochondrial biogenesis and function has been linked to aging, neurodegenerative diseases, and metabolic diseases such as type 2 diabetes and obesity [40]. Previous studies have documented in varied pathogenesis that the activation of PPAR*γ* and downstream proteins can induce improved mitochondrial function [41, 42]. Our study confirmed the protective effects of PPAR*γ*/PGC-1*α* against LPS-induced mitochondrial dysfunction in alveolar epithelial cells. Moreover, PPAR*γ* also contributes to reducing the impairment of MFN2 production during ALI, which is affected by the interactions between the ER and mitochondria and plays a key role in regulating mitochondrial dynamics.

Previous studies on macrophages and pancreatic *β*-cells have demonstrated that the activation of PPAR*γ* significantly attenuates ER stress [23, 34]. Our in vitro and in vivo studies in pulmonary alveolar cells also confirmed PPAR*γ*-dependent modulation of ER stress. Reduced PPAR*γ* is considered upstream of the enhanced ER stress [43]. However, activation of CHOP following ER stress can lead to the downregulation of PGC-1*α* and increased cell apoptosis [44]. These results indicate the existence of reverse signal transduction in the progression of cellular inflammatory response. Thus, an impaired balance between PPAR*γ*/PGC-1*α* and ER stress may play a role in exacerbating lung injury.

Obesity is also associated with lower APN levels. Studies have confirmed that APN expression in the lung tissue of obese mice is lower than that in normal mice [45]. APN is an adipokine with prominent anti-inflammatory properties [46]. It has been observed that mice with targeted deletion of the APN gene exhibited spontaneous activation of pulmonary vascular endothelial cells and were more susceptible to ALI [5]. In the present study, upregulation of APN was detected following the activation of PPAR*γ*, suggesting a potential role of adipokines in mediating PPAR*γ*-induced protection against ALI. A link between PPAR*γ* and APN has been proposed in previous studies. APN, a target gene of PPAR*γ*, is induced by PPAR-*γ* ligands via direct binding of the PPAR-*γ*/RXR heterodimer to the PPAR response elements (PPRE) in the APN promoter [47]. PPAR*γ* activation can regulate APN production at transcriptional and translational levels [48–50]. However, how they contribute to the anti-inflammation efficacy in the alveolar epithelia requires further investigation.

The present study has several limitations. First, A549 cells were used instead of primary alveolar type II cells obtained from obese mice. The A549 cell line is widely used as an in vitro model for type II pulmonary epithelial cells. Our study confirmed that the activation of PPAR*γ*/PGC-1*α* induces protective effects against LPS-induced epithelial injury by relieving ER stress and improving mitochondrial biosynthesis. However, the level of PPAR*γ* in the lung epithelia of obese mice and its changes during inflammation remain unclear. Second, although current evidence suggests that low levels of PPAR*γ* and APN in obese mice may contribute to susceptibility to ALI, it is not sufficient to demonstrate the impact of APN on ER stress and mitochondrial function. Further studies are required to elucidate the interactions between PPAR*γ*/PGC-1*α* and APN in mediating lung protection. Third, our experiments confirmed that PPAR*γ* activation has a preventive effect on ALI/ARDS in obese mice. But it is also important to determine the ability of PPAR*γ* activation in reverting a pre-existing inflammatory state in lung. Therefore, we will continue to study this issue in the future.

## 5. Conclusion

In conclusion, the present study demonstrated that obesity-induced downregulation of PPAR*γ* may increase susceptibility to ALI. The anti-inflammatory efficacy of PPAR*γ* in alveolar epithelia is mediated by the alleviation of ER stress and promotion of mitochondrial biogenesis.

## Figures and Tables

**Table 1:** Sequences of primer pairs used for amplification of mRNA by real-time PCR.

	Forward	Reverse
Mouse		
*β*-Actin	AGATTACTGCTCTGGCTCCTAGC	ACTCATCGTACTCCTGCTTGCT
PPAR*γ*	TTCAAGGGTGCCAGTTTCG	CCATCTTTATTCATCAGGGAGG
PGC-1*α*	TACAACAATGAGCCTGCGAAC	GCATCAAATGAGGGCAATCC
NRF-1	CATGATCCTGGAAGACCTCG	CCCGACCTGTGGAATACTTG
TFAM	GGAGGCAAAGGATGATTCGG	CTTCGTCCAACTTCAGCCATCT
MFN2	ATGGGCATTCTTGTGGTCG	GCTTCTCACTGGCGTATTCC
GRP78	AGCCAACTGTAACAATCAAGGTC	GCTGTCACTCGGAGAATACCAT
CHOP	AGGTCCTGTCCTCAGATGAAATTG	GGCTTTGGGATGTGCGTGT
Caspase12	TAGCCACTGCTGATACAGATGAG	AACCAGTCTTGCCTACCTTCC
APN	CGTCACTGTTCCCAATGTACC	GAGGCTCACCTTCACATCTTTC
TNF-*α*	TTCTCATTCCTGCTTGTGG	ACTTGGTGGTTTGCTACG
IL-1*β*	TTCCTTGTGCAAGTGTCTGAAG	CACTGTCAAAAGGTGGCATTT
Human		
*β*-Actin	AGAAAATCTGGCACCACACCT	GATAGCACAGCCTGGATAGCA
PPAR*γ*	AGCCCTTCACTACTGTTGACTTC	CTCAGAATAATAAGGTGGAGATGC
PGC-1*α*	ACACGAGGAAAGGAAGACCAAG	CCTGCCAATCAGAGGAGACATC
NRF-1	CTTCAGAATTGCCAACCACG	TGCTTGCGTCGTCTGGATG
TFAM	GCGTTTCTCCGAAGCATGTG	GCCAAGACAGATGAAAACCACC
MFN2	GCAGATTACGGAGGAAGTGGAG	TTGAGGACTACTGGAGAAGGGTG
GRP78	TCTGGGTACATTTGATCTGACTGG	CAGGCGATTCTGGTCATTGG
CHOP	ATCTTCACCACTCTTGACCCTG	GACCACTCTGTTTCCGTTTCC
Caspase12	AGCACCAGTCCTCAGACAGCA	GTGAACCAAACAATCCCAGCA
TNF-*α*	TCTTCTCCTTCCTGATCGTGG	AGGGCTGATTAGAGAGAGGTCC
IL-1*β*	GAAATGATGGCTTATTACAGTGGC	TTGCTGTAGTGGTGGTCGGAG

## Data Availability

All data used in this study are available from the corresponding author on request.

## References

[1] Arroyo-Johnson C., Mincey K. D. (2016). Obesity epidemiology worldwide. *Gastroenterology Clinics of North America*.

[2] Varban O. A., Cassidy R. B., Bonham A. (2017). Factors associated with achieving a body mass index of less than 30 after bariatric surgery. *JAMA Surgery*.

[3] Peters U., Suratt B. T., Bates J. H. T., Dixon A. E. (2018). Beyond BMI: obesity and lung disease. *Chest*.

[4] Manicone A. M., Gong K., Johnston L. K., Giannandrea M. (2016). Diet-induced obesity alters myeloid cell populations in naïve and injured lung. *Respiratory Research*.

[5] Konter J. M., Parker J. L., Baez E. (2012). Adiponectin attenuates lipopolysaccharide-induced acute lung injury through suppression of endothelial cell activation. *The Journal of Immunology*.

[6] Wei K., Luo J., Cao J., Peng L., Ren L., Zhang F. (2020). Adiponectin protects obese rats from aggravated acute lung injury via suppression of endoplasmic reticulum stress. *Diabetes, Metabolic Syndrome and Obesity: Targets and Therapy*.

[7] Kota B. P., Huang T. H., Roufogalis B. D. (2005). an overview on biological mechanisms of PPARs. *Pharmacological Research*.

[8] Hetzel M., Walcher D., Grüb M., Bach H., Hombach V., Marx N. (2003). Inhibition of MMP-9 expression by PPARgamma activators in human bronchial epithelial cells. *Thorax*.

[9] Arnold R., König W. (2006). Peroxisome-proliferator-activated receptor-gamma agonists inhibit the release of proinflammatory cytokines from RSV-infected epithelial cells. *Virology*.

[10] Standiford T. J., Keshamouni V. G., Reddy R. C. (2005). Peroxisome proliferator-activated receptor-{gamma} as a regulator of lung inflammation and repair. *Proceedings of the American Thoracic Society*.

[11] Chinetti G., Griglio S., Antonucci M. (1998). Activation of proliferator-activated receptors *α* and *γ* induces apoptosis of human monocyte-derived macrophages. *Biological Chemistry*.

[12] Chiang M. C., Cheng Y. C., Nicol C. J. (2015). Rosiglitazone activation of PPAR*γ*-dependent signaling is neuroprotective in mutant huntingtin expressing cells. *Cell Research*.

[13] Lehrke M., Lazar M. A. (2005). The many faces of PPARgamma. *Cell*.

[14] Spiegelman B. M., Flier J. S. (2001). Obesity and the regulation of energy balance. *Cell*.

[15] Sundararajan S., Jiang Q., Heneka M., Landreth G. (2006). PPAR*γ* as a therapeutic target in central nervous system diseases. *Neurochemistry International*.

[16] He J., Qi D., Tang X. M. (2019). Rosiglitazone promotes ENaC-mediated alveolar fluid clearance in acute lung injury through the PPAR*γ*/SGK1 signaling pathway. *Cellular & Molecular Biology Letters*.

[17] Reddy A. T., Lakshmi S. P., Reddy R. C. (2016). PPAR*γ*as a novel therapeutic target in lung cancer. *PPAR Research*.

[18] Li J., Dai A., Hu R., Zhu L., Tan S. (2010). Positive correlation between PPAR&gamma;/PGC-1&alpha; and &gamma;-GCS in lungs of rats and patients with chronic obstructive pulmonary disease. *Acta Biochimica et Biophysica Sinica*.

[19] Zieleniak A., Wojcik M., Wozniak L. A. (2008). Structure and physiological functions of the human peroxisome proliferator-activated receptor gamma. *Archivum Immunologiae et Therapiae Experimentalis*.

[20] Zhang H., Xu X., Chen L. (2011). Molecular determinants of magnolol targeting both RXR*α* and PPAR*γ*. *PLoS One*.

[21] Brust R., Shang J., Fuhrmann J. (2018). A structural mechanism for directing corepressor-selective inverse agonism of PPAR*γ*. *Nature Communications*.

[22] Zhu T., Zhang W., Feng S. J., Yu H. P. (2016). Emodin suppresses LPS-induced inflammation in RAW264.7 cells through a PPAR*γ*-dependent pathway. *Immunopharmacology*.

[23] Ikeda J., Ichiki T., Takahara Y. (2015). PPAR*γ* agonists attenuate palmitate-induced ER stress through up-regulation of SCD-1 in macrophages. *PLoS One*.

[24] Quintanilla R. A., Jin Y. N., Fuenzalida K., Bronfman M., Johnson G. V. W. (2008). Rosiglitazone treatment prevents mitochondrial dysfunction in mutant huntingtin-expressing cells. *Biological Chemistry*.

[25] Wu X., Luo J., Liu H. (2020). Recombinant adiponectin peptide promotes neuronal survival after intracerebral haemorrhage by suppressing mitochondrial and ATF4-CHOP apoptosis pathways in diabetic mice via Smad3 signalling inhibition. *Cell Proliferation*.

[26] Shah D., Torres C., Bhandari V. (2019). Adiponectin deficiency induces mitochondrial dysfunction and promotes endothelial activation and pulmonary vascular injury. *The FASEB Journal*.

[27] Hwang J. S., Kang E. S., Ham S. A. (2012). Activation of peroxisome proliferator-activated receptor *γ* by rosiglitazone inhibits lipopolysaccharide-induced release of high mobility group box 1. *Mediators of Inflammation*.

[28] Zhang H., You L., Zhao M. (2019). Rosiglitazone attenuates Paraquat-induced lung fibrosis in rats in a PPAR gamma-dependent manner. *European Journal of Pharmacology*.

[29] Wu D., Zhang H., Wu Q. (2021). Sestrin 2 protects against LPS-induced acute lung injury by inducing mitophagy in alveolar macrophages. *Life Sciences*.

[30] Chuang C. Y., Chen T. L., Cherng Y. G., Tai Y. T., Chen T. G., Chen R. M. (2011). Lipopolysaccharide induces apoptotic insults to human alveolar epithelial A549 cells through reactive oxygen species-mediated activation of an intrinsic mitochondrion-dependent pathway. *Toxicology*.

[31] Yang Y., Zhong Z. T., Xiao Y. G., Chen H. B. (2022). The activation of AMPK/NRF2 pathway in lung epithelial cells is involved in the protective effects of kinsenoside on lipopolysaccharide-induced acute lung injury. *Oxidative Medicine and Cellular Longevity*.

[32] Patel K. M., Wright K. L., Whittaker P. (2005). Differential modulation of COX-2 expression in A549 airway epithelial cells by structurally distinct PPAR(gamma) agonists: evidence for disparate functional effects which are independent of NF-(kappa)B and PPAR(gamma). *Cellular Signalling*.

[33] Ge L. N., Yan L., Li C., Cheng K. (2019). Bavachinin exhibits antitumor activity against non-small cell lung cancer by targeting PPAR*γ*. *Molecular Medicine Reports*.

[34] Hong S. W., Lee J., Cho J. H. (2018). Pioglitazone attenuates palmitate-induced inflammation and endoplasmic reticulum stress in pancreatic *β*-cells. *Endocrinology and Metabolism*.

[35] Lin C. H., Nicol C. J. B., Cheng Y. C. (2018). Rosiglitazone rescues human neural stem cells from amyloid-beta induced ER stress via PPAR*γ* dependent signaling. *Cell Research*.

[36] Puigserver P., Wu Z., Park C. W., Graves R., Wright M., Spiegelman B. M. (1998). A cold-inducible coactivator of nuclear receptors linked to adaptive thermogenesis. *Cell*.

[37] Hondares E., Mora O., Yubero P. (2006). Thiazolidinediones and rexinoids induce peroxisome proliferator-activated receptor-coactivator (PGC)-1alpha gene transcription: an autoregulatory loop controls PGC-1alpha expression in adipocytes via peroxisome proliferator-activated receptor-gamma coactivation. *Endocrinology*.

[38] Park S. S., Lee Y. J., Kang H. (2019). Lactobacillus amylovorus KU4 ameliorates diet-induced obesity in mice by promoting adipose browning through PPAR*γ* signaling. *Scientific Reports*.

[39] Liu L., Zhang T., Hu J. (2020). Adiponectin/SIRT1 Axis induces white adipose browning after vertical sleeve gastrectomy of obese rats with type 2 diabetes. *Obesity Surgery*.

[40] Popov L. D. (2020). Mitochondrial biogenesis: an update. *Cell. Mol. Med.*.

[41] Yeh J. H., Wang K. C., Kaizaki A. (2021). Pioglitazone ameliorates lipopolysaccharide-induced behavioral impairment, brain inflammation, white matter injury and mitochondrial dysfunction in neonatal rats. *International Journal of Molecular Sciences*.

[42] Chiang M. C., Nicol C. J., Cheng Y. C., Lin K. H., Yen C. H., Lin C. H. (2016). Rosiglitazone activation of PPAR*γ*-dependent pathways is neuroprotective in human neural stem cells against amyloid-beta-induced mitochondrial dysfunction and oxidative stress. *Aging*.

[43] Yun C. W., Han Y. S., Lee S. H. (2019). PGC-1*α* controls mitochondrial biogenesis in drug-resistant colorectal cancer cells by regulating endoplasmic reticulum stress. *International Journal of Molecular Sciences*.

[44] Chen X., Zhong J., Dong D., Liu G., Yang P. (2017). Endoplasmic reticulum stress-induced CHOP inhibits PGC-1*α* and causes mitochondrial dysfunction in diabetic embryopathy. *Toxicological Sciences*.

[45] Zhu L., Chen X., Chong L. (2019). Adiponectin alleviates exacerbation of airway inflammation and oxidative stress in obesity-related asthma mice partly through AMPK signaling pathway. *Immunopharmacology*.

[46] Tumminia A., Vinciguerra F., Parisi M. (2019). Adipose tissue, obesity and adiponectin: role in endocrine cancer risk. *International Journal of Molecular Sciences*.

[47] Iwaki M., Matsuda M., Maeda N. (2003). Induction of adiponectin, a fat-derived antidiabetic and antiatherogenic factor, by nuclear receptors. *Diabetes*.

[48] Maeda N., Takahashi M., Funahashi T. (2001). PPARgamma ligands increase expression and plasma concentrations of adiponectin, an adipose-derived protein. *Diabetes*.

[49] Banga A., Unal R., Tripathi P. (2009). Adiponectin translation is increased by the PPARgamma agonists pioglitazone and omega-3 fatty acids. *American Journal of Physiology-Endocrinology and Metabolism*.

[50] Tao L., Wang Y., Gao E. (2010). Adiponectin. *Circulation Research*.

